# A semi-quantitative GeLC-MS analysis of temporal proteome expression in the emerging nosocomial pathogen *Ochrobactrum anthropi*

**DOI:** 10.1186/gb-2007-8-6-r110

**Published:** 2007-06-13

**Authors:** Robert Leslie James Graham, Mohit K Sharma, Nigel G Ternan, D Brent Weatherly, Rick L Tarleton, Geoff McMullan

**Affiliations:** 1 School of Biomedical Sciences, University of Ulster, Coleraine, County Londonderry BT52 1SA, UK; 2 The Center for Tropical and Emerging Global Diseases, University of Georgia, Athens, GA 30605, USA

## Abstract

**Background:**

The α-Proteobacteria are capable of interaction with eukaryotic cells, with some members, such as *Ochrobactrum anthropi*, capable of acting as human pathogens. *O. anthropi *has been the cause of a growing number of hospital-acquired infections; however, little is known about its growth, physiology and metabolism. We used proteomics to investigate how protein expression of this organism changes with time during growth.

**Results:**

This first gel-based liquid chromatography-mass spectrometry (GeLC-MS) temporal proteomic analysis of *O. anthropi *led to the positive identification of 131 proteins. These were functionally classified and physiochemically characterized. Utilizing the emPAI protocol to estimate protein abundance, we assigned molar concentrations to all proteins, and thus were able to identify 19 with significant changes in their expression. Pathway reconstruction led to the identification of a variety of central metabolic pathways, including nucleotide biosynthesis, fatty acid anabolism, glycolysis, TCA cycle and amino acid metabolism. In late phase growth we identified a number of gene products under the control of the *oxyR *regulon, which is induced in response to oxidative stress and whose protein products have been linked with pathogen survival in response to host immunity reactions.

**Conclusion:**

This study identified distinct proteomic profiles associated with specific growth points for *O. anthropi*, while the use of emPAI allowed semi-quantitative analyses of protein expression. It was possible to reconstruct central metabolic pathways and infer unique functional and adaptive processes associated with specific growth phases, thereby resulting in a deeper understanding of the physiology and metabolism of this emerging pathogenic bacterium.

## Abstract 2

A semi-quantitative gel-based analysis identifies distinct proteomic profiles associated with specific growth points for the nosocomial pathogen *Ochrobactrum anthropi*.

## Background

The α-Proteobacteria are a biologically diverse group with many members capable of interaction with eukaryotic cells and able to function as intracellular symbionts or as pathogens of plants and animals. Some members are important human pathogens, some can establish asymptomatic chronic animal infections, and others are agriculturally important, assisting plants with nitrogen fixation [1]. The α-2 subgroup of the Proteobacteria contain the well-known genera *Rhizobacteria*, *Agrobacterium*, *Rickettsia*, *Bartonella *and *Brucella*, which include species of widespread medical and agricultural importance [2]. A less well known member of this group is the genus *Ochrobactrum*, which is genetically most closely related to the genus *Brucella *[3].

Until 1998, *Ochrobactrum anthropi *was considered to be both the sole and type species of the genus *Ochrobactrum*, despite the genetic and phenotypic heterogeneity visible within isolates of the species [4]. Subsequent analysis by Velasco *et al*. [5] resulted in the description of *O. intermedium *as a second species. Two new species, *O. grignonense *and *O. tritici*, were isolated from soil and wheat rhizoplane systems by Lebuhn *et al*. [6], and most recently, *O. gallinifaecis *was isolated from a chicken fecal sample, *O. cystisi *from nodules of *Cystisus scoparius *and *O. pseudintermedium *from clinical isolates [7,8].

*Ochrobactrum *species have been described as being environmentally abundant free-living α-Proteobacteria. A number of reports exist in the literature describing the use of *Ochrobactrum *species as either a source of biotechnologically useful enzymes [9-11] or in the detoxification of xenobiotic compounds such as halobenzoates [12-16]. The ability of *Ochrobactrum *species to act as legume endosymbionts in temperate genera such as *Lupinus*, *Musa *and *Acacia *has also recently been demonstrated [17-19].

*O. anthropi *has been identified in clinical samples [20] and has been the cause of a growing number of hospital-acquired infections usually, but not always, in immunocompromised hosts [21-25]. The organism has been found to adhere, possibly as a result of biofilm formation, to the surface of catheters, pacemakers, intraocular lenses and silicon tubing, thus representing potential sources of infection in the clinical environment [26,27]. Upon infection, *O. anthropi *has been shown to cause pancreatic abscess, catheter-related bacteremia, endophthalmitis, urinary tract infection and endocarditis [21]. *O. anthropi *strains usually are resistant to all β-lactams, with the exception of the antibiotic imipenem. Nadjar and co-workers [20] demonstrated that in at least one isolate, such resistance was due to an extended spectrum β-lactamase. Other than imipenem, the most effective antimicrobial agents for treating human infection that have thus far been reported are trimethoprim-sulfamethoxazole and ciprofloxacin [23,24].

As with its closest genetically related genus, *Brucella*, the genomes of *O. intermedium *and *O. anthropi *are composed of two independent circular chromosomes [28]. Recent work by Teyssier *et al*. [29] revealed an exceptionally high level of genomic diversity within *Ochrobactrum *species, possibly reflecting their adaptability to various ecological niches. Whilst there is currently no publicly available genome sequence data for any *Ochrobactrum *species, genome information does exist for 20 α-Proteobacteria species, including four species of *Brucella*. The availability of such information not only offers an excellent model system to study the forces, mechanisms and rates by which bacterial genomes evolve [30] but also to carry out functional genomic and proteomic investigations of these and closely related organisms.

Beynon [31] identified a number of phases in the proteomic study of an organism or disease process. In the initial 'identification' phase, scientists are predominantly concerned with gaining insight into the identities of proteins present within the system with which they are working. Recently, we reported such a study of the soluble sub-proteome of *O. anthropi *[32]. This allowed the identification of 249 proteins involved in a variety of essential cellular pathways, including nucleic acid, amino and fatty acid anabolism and catabolism, glycolysis, TCA cycle, pyruvate and selenoamino acid metabolism. In addition, we identified a number of potential virulence factors of relevance to both plant and human disease. This previous study is a valuable reference point for the proteome of this emerging pathogen. These types of 'identification' studies, whilst useful, tell us very little about the functional role of these proteins within cellular networks. Further developmental phases were described by Beynon [31], including 'characterization' proteomics, and finally 'quantitative' proteomics in which the emphasis is on the pair-wise comparison of two proteomes and the quantifying of specific proteins present. To develop further our understanding of *O. anthropi *we have performed a comparative and semiquantitative proteomic analysis to identify the temporal changes in expression and abundance of proteins during growth of this organism. The soluble sub-proteome of *O. anthropi *grown aerobically in nutrient broth was compared at early phase and late phase growth, with 19 proteins having significant changes in their observed expression. Pathway reconstruction analysis was carried out and led to the identification of a variety of core metabolic processes, thus giving insights into the underlying physiology and biochemistry of this organism. During the late phase of growth of *O*.*anthropi *a number of gene products normally induced in response to oxidative stress were identified. These expressed gene products, part of the *OxyR *regulon, have been linked with pathogen survival in the host environment.

## Results and discussion

### Comprehensive analysis of the *O. anthropi *soluble sub-proteome

In this study we report the first gel based comparative proteomic analysis of the α-Proteobacterium *O. anthropi *at two distinct phases of growth. This multidimensional analysis involved the soluble sub-proteome being first separated by one-dimensional PAGE. The resultant gel was then cut into nine fractions based on the SeeBlue™ Plus 2 molecular mass markers. Each gel fraction was then trypsinized and the extracted peptides separated on a reversed phase C_18 _column over a 60 minute time period prior to being introduced onto the mass spectrometer. This methodology allowed the identification of a total of 131 proteins from the soluble sub-proteome under the two growth phases. This expressed gene product subset represents an estimated 3% of the total *O. anthropi *proteome, employing data based upon the typical predicted genome size [29]. No data are currently available in the literature on the expected distribution of proteins within sub-proteomic fractions of *O. anthropi*. As a benchmark, however, a study concentrating mainly on the analysis of the cytosolic proteins of *Brucella melitensis *16M, a phylogenetically closely related organism, identified 187 proteins equating to 6% of its predicted proteome [33,34].

As previously reported, [35] due to the complex nature of the peptide mixtures to be analyzed, the separation capabilities of the liquid chromatography (LC)-mass spectrometry (MS) systems are often exceeded. In this study all peptide fractions were analyzed three separate times in order to increase overall peptide identifications. In the current study, automated curation of our initial dataset by the heuristic bioinformatic tool PROVALT [36], along with manual curation, led to the positive identification of 89 proteins at early phase and 95 proteins at late phase growth.

### Characterisation of the *O. anthropi *soluble sub-proteome at early and late phase growth

**Figure 1:**
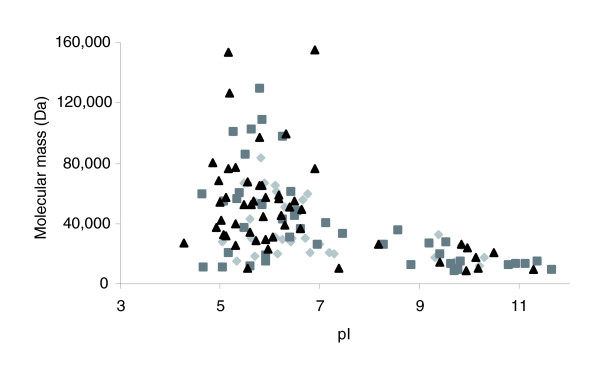
Theoretical two-dimensional map of the soluble sub-proteome of *O. anthropi*. Diamonds, early growth phase; squares, both growth conditions; triangles, late growth phase.

Within the protein subset identified from the soluble sub-proteome, 34 proteins were uniquely identified in the early phase of growth, 55 proteins were found under both growth conditions and 40 were found to be unique to the later growth phase. The identified proteins had a wide range of physio-chemical properties in respect to pI and molecular mass (M_r_) (Figure 1). This two-dimensional visualization showed that the smallest protein identified in early growth was the 30S ribosomal protein S17 (M_r _= 9,123 Da) whilst at the late growth condition it was the cold shock protein CSPA (M_r _= 8,963 Da). The largest protein identified under both conditions was DNA directed RNA polymerase beta chain (M_r _= 153,688 Da). The most acidic protein identified under both conditions was the 30S ribosomal protein S1 (pI = 4.28) while the most basic in the early growth condition was the 30S ribosomal protein S5 (pI = 10.49) and in the late growth condition was the 30S ribosomal protein S20 (pI = 11.63).

Proteins identified within the two growth conditions were quantified using the Exponentially Modified Protein Abundance Index (emPAI) and can be seen in Table 1 (for those proteins unique to early phase growth), Table 2 (for those proteins common to both growth conditions) and Table 3 (for those proteins unique to late phase growth) [37]. This method allows the quantification of individual identified proteins by utilizing database and Mascot output information, in order to give an emPAI value. The emPAI value can then be used to estimate the protein content within the sample mixture in molar fraction percentages. In addition, the fold change in expression level of proteins identified under both growth conditions can be estimated, thus giving further insights into cellular processes. The most abundant protein as calculated by molar fraction percentages under both conditions was the 30S ribosomal protein S1 (Table 2). The least abundant protein under early growth conditions was 30S ribosomal protein S17 (Table 1) and under late phase growth conditions was Valyl-tRNA synthetase (Table 3).

**Table 1:** Proteins identified in early growth phase with their bioinformatic analysis and emPAI calculation

Accession no. (NCBI)	Protein	Mowse	PSortB		SignalP SP	SecP	emPAI	Protein (M%)	Species
								
			L	SP					
17984580	GTP-binding tyrosine phosphorylated protein	189	C	No	No	No	0.112	0.442	Bm
17982767	30S ribosomal protein S2	158	C	No	No	No	0.199	0.785	Bm
17983035	Glutamyl-tRNA amidotransferase, beta subunit	145	C	No	No	No	0.117	0.461	Bm
17984058	Phenylalanyl-tRNA synthetase beta subunit	141	C	No	No	No	0.079	0.311	Bm
17982501	UDP-N-acetylmurate - alanine ligase (cytoplasmic peptidoglycan synthetase	128	C	No	No	No	0.104	0.410	Bm
17984007	3-Oxoacyl-(acyl-carrier-protein) synthase 1	110	C	No	N0	No	0.186	0.733	Bm
17982216	Hypothetical cytosolic protein	109	C	No	No	Y 0.69	0.138	0.544	Bm
17982947	Methionyl-tRNA synthetase	101	C	No	YHA-LL^14,15^	No	0.050	0.197	Bm
17982718	Adenylate kinase	99	C	No	No	No	0.178	0.702	Bm
17984859	Glutamyl-tRNA amidotransferase, alpha subunit	87	U	No	No	No	0.178	0.702	Bm
17984546	Piperideine-6-carboxylate dehydrogenase	85	C	No	No	No	0.076	0.300	Bm
17982155	Branched chain amino acid ABC transporter, periplasmic AA binding protein	83	P	No	No	No	0.274	1.080	Bm
17982770	Ribosome recycling factor	82	C	No	No	No	0.130	0.513	Bm
17983887	Dihydroxy-acid dehydratase	80	C	No	AGA-AG^20,21^	No	0.074	0.292	Bm
17982681	Transcription antitermination protein nusG	77	U	No	No	No	0.186	0.733	Bm
17983656	Glucose-6-phosphate isomerase	74	U	No	No	No	0.084	0.331	Bm
17984871	Glucosamine-fructose-6-phosphate aminotransferase (isomerizing)	74	C	No	No	No	0.151	0.595	Bm
17982453	Hypothetical protein (immunoreactive 28 kDa omp)	69	P	No	AFA-QE^28,29^	Y 0.9	0.138	0.544	Bm
17740384	30S ribosomal protein S8	66	C	No	No	No	0.096	0.379	At
17983241	Nucleoside diphosphate kinase	64	C	No	No	No	0.156	0.615	Bm
17983005	ABC transporter ATP-binding protein	63	U	No	No	No	0.064	0.252	Bm
17982925	NAD-dependent malic enzyme, malic oxidoreductase	62	U	No	No	No	0.067	0.262	Bm
17983949	3-Deoxy-manno-oculosonate cytidylyltransferase	62	C	No	ANG-YI^28,29^	No	0.052	0.205	Bm
17983146	30S ribosomal protein S9	60	U	No	No	Y 0.70	0.146	0.576	Bm
17982830	Single-stranded DNA binding protein	59	U	No	No	Y 0.82	0.172	0.678	Bm
17982823	ATP-dependent Clp protease proteolytic subunit	58	C	No	No	No	0.233	0.919	Bm
17984491	Lipoprotein (ABC transporter substrate binding protein)	57	U	Yes	SHA-ED^37,38^	No	0.076	0.300	Bm
17982653	Methionine aminopeptidase	56	C	No	No	No	0.117	0.461	Bm
17984405	GTP-binding protein LepA	51	C	No	No	No	0.057	0.225	Bm
49238170	2-Dehydro-3-deoxyphosphooctonate aldolase	51	C	No	No	No	0.138	0.544	Bh
17982695	30S ribosomal protein S10	50	C	No	No	No	0.194	0.765	Bm
27353255	Transriptional regulatory protein	47	U	No	SHS-DR^12,13^	No	0.096	0.379	Bj
86284664	ABC transporter ATP-binding	42	CM	No	No	No	0.102	0.402	Re
17984791	Branched chain amino acid ABC aminotransferase	40	C	No	No	No	0.210	0.828	Bm

Cellular localizations: C, cytoplasmic; CM, cytoplasmic membrane; E, extracellular; P, periplasmic; U, unknown. SecP, SecretomeP; SP, signal peptide. Species: At, *Agrobacterium tumefaciens*; Ba, *Brucella abortus*; Bh, *Bartonella henselae*; Bj, *Bradyrhizobium japonicum*; Bm, *Brucella melitensis*; Bs, *Brucella suis*; Re, *Rhizobium etli*.

**Table 2:** Proteins identified in both growth phases with their bioinformatic analysis and emPAI calculation

Accession no. (NCBI)	Protein	Mowse		PSortB		SignalP	SecP	emPAI		Protein (M%)		Fold change	Species
							
		0.3	1.2	L	SP	SP		0.3	1.2	0.3	1.2		
17985267	60 kDa chaperonin GroEl	1334	1734	C	No	No	No	0.778	0.884	3.068	2.985	1.0	Bm
17982679	Protein translation elongation factor Tu	828	1133	C	No	AMA-KS^17,18^	No	0.897	1.153	3.537	3.893	0.9	Bm
17982693	Protein translation elongation factor G	547	884	C	No	No	No	0.459	0.503	1.810	1.698	1.1	Bm
17982686	DNA directed RNA polymerase beta chain	601	686	C	No	No	No	0.211	0.183	0.832	0.618	1.3	Bm
17982688	DNA directed RNA polymerase beta' chain	461	675	C	No	No	No	0.132	0.172	0.520	0.581	0.9	Bm
17984056	DNAK protein (HSP 70)	404	613	C	No	No	Y 0.69	0.225	0.288	0.887	0.972	0.9	Bm
17982961	30S ribosomal protein S1	541	611	U	No	No	Y 0.85	4.623	3.645	18.228	12.308	1.5	Bm
17983895	Aconitate hydratase	288	563	C	No	No	No	0.18	0.297	0.710	1.033	0.7	Bm
17981970	Electron transfer flavoprotein beta subunit	396	342	U	No	No	Y 0.63	0.469	0.469	1.849	1.584	1.2	Bm
17982110	Membrane-bound lytic murien transglycosylase B	238	103	CM	No	No	No	0.469	0.202	1.849	0.682	2.7	Bm
17984018	N utilization protein NusA	75	135	C	No	No	No	0.096	0.167	0.379	0.564	0.7	Bm
17982394	Ribose-phosphate pyrophosphokinase	95	192	U	No	No	No	0.146	0.250	0.576	0.844	0.7	Bm
17982015	Malate dehydrogenase	174	409	C	No	TLA-HL^25,26^	No	0.291	0.816	1.147	2.755	0.4	Bm
17982340	Periplasmic dipeptide transport protein pre	323	371	P	No	ASA-KT^37,38^	Y 0.93	0.39	0.51	1.538	1.722	0.9	Bm
17982978	Fumarate hydratase class I aerobic	301	309	C	No	No	No	0.406	0.291	1.601	0.983	1.6	Bm
17982732	Isocitrate dehydrogenase (NADP)	275	396	U	No	No	No	0.241	0.333	0.950	1.124	0.8	Bm
17982121	Phosphoribosylaminoimidazolecarboxamide formyltransferase	261	365	C	No	No	No	0.216	0.315	0.852	1.064	0.8	Bm
17983182	Aspartyl-tRNA synthetase	262	334	C	No	No	No	0.156	0.197	0.615	0.665	0.9	Bm
17982205	Transketolase	252	213	C	No	KAA-DG^16,17^	No	0.222	0.143	0.875	0.483	1.8	Bm
17982204	Glyceraldehyde 3-phosphate dehydrogenase	230	288	C	No	No	No	0.291	0.377	1.147	1.273	0.9	Bm
17983520	Enoyl-(acyl carrier protein) reductase (NADH)	232	216	C	No	No	No	0.648	0.493	2.555	1.665	1.5	Bm
17984008	Enoyl-(acyl carrier protein) reductase (NADH)	202	197	C	No	No	No	0.422	0.556	1.664	1.877	0.9	Bm
17982437	Carbamoyl-phosphate synthase large chain	125	286	U	Yes	No	No	0.038	0.161	0.150	0.544	0.3	Bm
17983107	30S ribosomal protein S4	206	62	U	No	No	Y 0.54	0.358	0.107	1.412	0.361	3.9	Bm
17982692	30S ribosomal protein S7	81	225	U	No	No	No	0.167	0.358	0.658	1.209	0.5	Bm
17985266	10 kDa chaperonin GroES	192	168	C	No	No	No	0.368	0.368	1.451	1.243	1.2	Bm
23463995	Conserved hypothetical protein	94	225	C	No	No	No	0.146	0.403	0.576	1.361	0.4	Bs
86279873	Polyribonucleotide nucleotidyltransferase protein	190	146	C	No	No	No	0.114	0.114	0.450	0.385	1.2	Re
17983037	Trigger factor, peptidylprolyl isomerase	131	224	C	No	No	No	0.089	0.119	0.351	0.408	0.9	Bm
17982138	ATP synthase F1, alpha chain	158	112	U	No	No	No	2.63	2.63	10.370	8.880	1.2	Bm
17982141	ATP synthase F1, beta chain	180	173	U	No	AEA-KP^15,16^	No	0.233	0.169	0.919	0.571	1.6	Bm
17984734	Glycine dehydrogenase (decarboxylating)	99	218	C	No	No	No	0.064	0.127	0.252	0.429	0.6	Bm
17982133	Transaldolase	179	218	U	No	No	No	0.374	0.374	1.475	1.263	1.2	Bm
17982713	30S ribosomal protein S5	164	133	C	No	No	No	0.138	0.089	0.544	0.301	1.8	Bm
17982705	30S ribosomal protein S17	96	205	U	No	No	Y 0.73	0.025	0.374	0.099	1.263	0.1	Bm
17983483	Malonyl coa-acyl carrier protein transacylase	160	135	C	No	No	No	0.259	0.259	1.021	0.875	1.2	Bm
17982471	ABC transporter ATP-binding protein YjjK	84	185	CM	No	No	No	0.084	0.114	0.331	0.385	0.9	Bm
17984086	Adenosylhomocysteinase	158	159	C	No	No	No	0.161	0.161	0.635	0.544	1.2	Bm
17983095	Phosphoribosylaminoimidazole-succinocarboxamide synthase	157	166	C	No	No	No	0.321	0.23	1.266	0.777	1.6	Bm
17982017	Succinyl-CoA synthetase alpha chain	155	176	C	No	No	No	0.291	0.291	1.147	0.983	1.2	Bm
17982721	DNA directed RNA polymerase alpha chain	134	65	C	No	No	No	0.211	0.138	0.832	0.466	1.8	Bm
17982016	Succinyl-CoA synthetase beta chain	76	157	C	No	No	No	0.094	0.197	0.371	0.665	0.6	Bm
17983100	Phosphoribosylformylglycinamidine synthase II	119	150	U	No	No	No	0.13	0.13	0.513	0.439	1.2	Bm
17982700	30S ribosomal protein S19	92	150	U	No	No	Y 0.9	0.291	0.469	1.147	1.584	0.7	Bm
17983486	30S ribosomal protein S18	119	63	U	No	No	Y 0.53	0.197	0.091	0.777	0.307	2.5	Bm
17982938	Glutamine synthetase I	115	63	C	No	No	Y 0.63	0.104	0.104	0.410	0.351	1.2	Bm
23463708	GMP synthase (glutamine-hydrolyzing)	113	87	C	No	No	No	0.227	0.107	0.895	0.361	2.5	Bs
17982702	30S ribosomal protein S3	57	136	C	No	No	No	0.045	0.143	0.177	0.483	0.4	Bm
17982781	Citrate synthase	112	114	C	No	No	No	0.146	0.146	0.576	0.493	1.2	Bm
17983059	Arginyl-tRNA synthetase	111	113	C	No	No	No	0.057	0.057	0.225	0.192	1.2	Bm
17982196	Hypothetical cytosolic protein	102	73	U	No	No	Y 0.69	0.14	0.069	0.552	0.233	2.4	Bm
17982768	Protein translation elongation factor Ts	99	132	C	No	No	No	0.067	0.138	0.264	0.466	0.6	Bm
17983768	Aldehyde dehydrogenase	41	111	C	No	No	No	0.089	0.138	0.351	0.466	0.8	Bm
17982113	Chorismate mutase	76	82	U	No	No	No	0.39	0.39	1.538	1.317	1.2	Bm
17983157	Integration host factor alpha subunit	57	102	U	No	No	Y 0.51	0.089	0.186	0.351	0.628	0.6	Bm

Cellular localizations: C, cytoplasmic; CM, cytoplasmic membrane; E, extracellular; P, periplasmic; U, unknown. SecP, SecretomeP; SP, signal peptide. Species: Bm, *Brucella melitensis*; Bs, *Brucella suis*; Re, *Rhizobium etli*.

**Table 3:** Proteins identified in late growth phase with their bioinformatic analysis and emPAI calculation

Accession no. (NCBI)	Protein	Mowse	PSortB		SignalP	SecP	emPAI	Protein (M%)	Species
								
			L	SP	SP				
17984094	Phosphoenol pyruvate carboxylase (ATP)	468	U	No	No	No	0.300	1.013	Bm
17983911	Arginosuccinate synthase	313	C	No	No	No	0.276	0.932	Bm
17982698	50S ribosomal protein L23	273	U	No	No	Y 0.5	0.097	0.327	Bm
17983035	Glutamyl-tRNA(GLN) amidotransferase subunit B	263	C	No	No	No	0.167	0.557	Bm
17982203	Phosphoglycerate kinase	178	C	No	No	No	0.239	0.807	Bm
17984924	Periplasmic oligopeptide-binding protein precursor	176	P	No	No	Y 0.89	0.183	0.618	Bm
17982826	DNA-binding protein HU alpha	170	U	No	LVA-AV^10,11^	Y 0.95	0.469	1.584	Bm
17982691	30S ribosomal protein S12	169	U	No	No	Y 0.83	0.291	0.983	Bm
17982154	Leucine, isoleucine, valine, threonine and alanine binding protein	157	P	Yes	AWA-DV^28,29^	Y 0.95	0.194	0.655	Bm
17984006	3-Hydroxydecanoyl-(acyl-carrier-protein) dehydratase	149	C	No	No	No	0.626	2.114	Bm
17983192	General L-amino acid-binding periplasmic protein AAPJ precursor	132	P	Yes	ASA-DT^24,25^	Y 0.65	0.225	0.760	Bm
17984058	Phenylalanyl-tRNA synthetase beta chain	131	C	No	No	No	0.054	0.182	Bm
17984780	N-methylhydantoinase (ATP-hydrolising) 5-oxoprolinase(EC3.5.2.9)	125	C	No	No	No	0.081	0.274	Bm
17983089	Adenylosuccinate lyase	120	C	No	No	No	0.086	0.290	Bm
17983993	30S ribosomal protein S20	120	U	No	No	Y 0.58	0.161	0.544	Bm
17983794	Hypothetical protein	119	C	No	No	No	0.469	1.584	Bm
17983437	Pyruvate, phosphate dikinase	112	C		No	No	0.047	0.159	Bm
17982937	Nitrogen regulatory protein P-II	108	C	No	No	No	0.233	0.789	Bm
17984078	Thioredoxin C-1	108	C	No	No	Y 0.84	0.291	0.983	Bm
17983171	Serine hydroxymethyltransferase	103	C	No	No	No	0.167	0.564	Bm
17984416	2,3,4,5-Tetrahdropyridine-2-carboxylate N-succinyltransferase	101	C	No	No	No	0.122	0.412	Bm
17984012	30S ribosomal protein S15	95	U	No	No	Y 0.54	0.069	0.233	Bm
17983482	Short-chain dehydrogenase	92	C	No	No	No	0.194	0.655	Bm
49238135	3-Oxoacyl-(acyl carrierprotein) reductase	92	C	No	No	No	0.076	0.257	Bh
17982682	50S ribosomal protein L11	91	U	No	AGA-AN^17,18^	Y 0.95	0.194	0.655	Bm
17984753	Alkyl hyroperoxide reductase C22 protein	85	C	No	No	No	0.274	0.925	Bm
17982411	Cold shock protein CSPA	82	C	No	No	Y 0.81	0.584	1.972	Bm
17983290	Dihydrodipicolinate synthase	82	C	No	ITA-LV^22,23^	No	0.122	0.412	Bm
17982131	Leucyl-tRNA synthetase	77	C	No	No	No	0.023	0.078	Bm
86283673	Dipeptide ABC transporter, substrate binding	75	P	Yes	AFA-ET^31,32^	Y 0.91	0.072	0.243	Re
17982712	50s ribosomal protein L18	74	U	No	No	No	0.072	0.243	Bm
23347767	Valyl-tRNA synthetase	73	C	No	No	No	0.019	0.064	Bs
17982719	30S ribosomal protein S13	72	C	No	No	No	0.072	0.243	Bm
17983459	Thiol peroxidase	69	U	No	No	Y 0.89	0.122	0.412	Bm
17982531	Hypothtical cytosolic protein	68	C	No	No	No	0.186	0.628	Bm
17984569	Osmotically inducible protein C	68	U	No	No	Y 0.82	0.069	0.233	Bm
17981953	Histidinol-phosphate aminotransferase	66	C	No	No	No	0.067	0.226	Bm
15073728	Probable isoleucyl-tRNA synthetase protein	64	C	No	No	No	0.038	0.128	Sm
17984859	Glutamyl-tRNA(GLN) amidotransferase subunit A	63	U	No	No	No	0.109	0.368	Bm
17984521	Urocanate hydratase	57	U	No	No	No	0.069	0.233	Bm

Cellular localizations: C, cytoplasmic; CM, cytoplasmic membrane; E, extracellular; P, periplasmic; U, unknown. SecP, SecretomeP; SP, signal peptide. Species: Bm, *Brucella melitensis*; Bs, *Brucella suis*; Re, *Rhizobium etli*; Sm, *Sinorhizobium meliloti*.

Proteomic analysis of the origin of the identified proteins in this study supports previous genomic studies showing that, phylogentically, the genus *Ochrobactrum *is most closely related to *Brucella*, with 93.9% of the proteins identified having closest match to this genus. The remaining proteins were matched to other members of the α-2 subgroup of the Proteobacteria (*Rhizobacteria *(3.8%), *Bartonella *(1.5%) and *Agrobacterium *(0.8%)).

**Figure 2:**
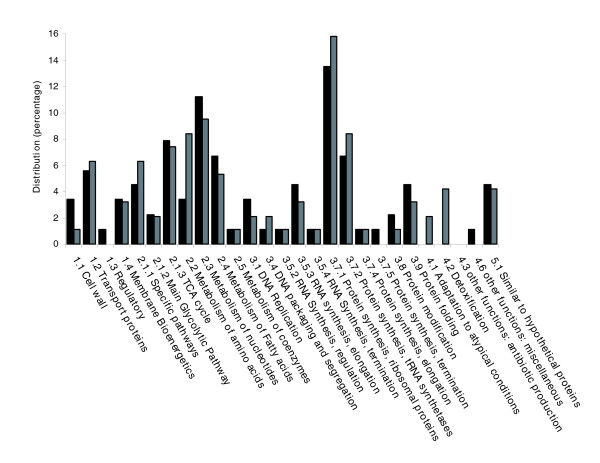
Functional categorisation of identified proteins from the soluble sub-proteome of *O. anthropi*. Gray bars, early growth phase; black bars, late growth phase.

Of the 131 proteins detected in this study, functional roles for 125 proteins (95.4%) were known or could be predicted from database analysis. Proteins within this soluble sub-proteome were assigned to functional categories utilizing methodologies as previously described by Takami *et al*. [38] and Wasinger *et al*. [39]. Figure 2 shows that proteins of the largest category of identified proteins under both growth conditions were involved in protein synthesis (ribosomal proteins), followed by those involved in metabolism of nucleotides and nucleic acids, then those involved in metabolism of amino acids and related molecules. The remaining proteins were distributed amongst the other functional categories. The functional categories of Metabolism of nucleotides, DNA replication, RNA synthesis (elongation), Protein modification and Protein folding are found to be present at higher levels in early growth phase compared to late phase growth. In the late phase of growth, Transport proteins, Specific pathways, Metabolism of amino acids, Protein synthesis (ribosomal proteins) and Protein synthesis (tRNA synthetases) are better represented. Furthermore, the late growth phase was the only one to have proteins present from the Adaptation to atypical conditions (2.1%) and Detoxification (4.2%) functional categories. It is worth noting that assignment of proteins to functional categories is complicated, as exemplified in the case of the Metabolism of nucleotides category, by the anaplerotic nature of bacterial enzymes with a number of proteins that could also have been classified within the Metabolism of amino acids category.

The rapid increase in genomic data over the past decade has revealed many important aspects of microbial cellular processes; however, there are still a significant number of potential gene products for which we know nothing, save that they are classified as 'hypothetical proteins'. Indeed, within the genome sequence of *B. melitensis *strain 16M, the closest relative phylogenetically of *O. anthropi *for which genomic data are available, some 716 predicted gene products, equivalent to 22% of the total genome, are predicted to be either hypothetical proteins or proteins of unknown function. In previous work we have underlined the necessity to assign, where possible, an element of biological functionality to such gene products in order to develop both systems biology and our understanding of cellular processes within these organisms. Within the current study we have established the presence of six proteins that had previously been annotated as hypothetical conserved proteins. The identification of such proteins within the cell-extract of *O. anthropi *establishes the biological functionality of these 'hypothetical' predicted protein coding sequences, and once more elegantly demonstrates the potential of proteomics to validate bioinformatics predictions.

Having established the presence of such proteins and wishing to understand how they contribute to functional processes, we further examined them using NCBI BLASTp [40]. Such an approach allows conserved domains within protein sequences to be identified and thereby enables a degree of inferred functionality. Using this methodology, however, allowed us to assign putative function to only one of these proteins, NCBI:23463995. The search identified two conserved domains, pfam 01480, GFO_IDH_MocA; Oxidoreductase family involved in utilization of NADP or NAD and COG 1748; Saccharopine dehydrogenase and related proteins involved in amino acid transport and metabolism.

### Sub-cellular protein localization

**Figure 3:**
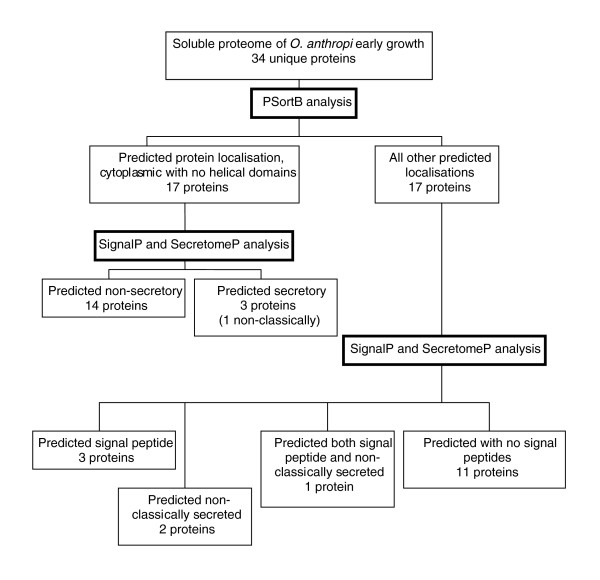
Overview of identified proteins from the soluble sub-proteome of *O. anthropi *at the early growth phase. Cellular localization was predicted based upon the use of PSortB v2.0.4 [41,42], SignalP v3.0 [43,44], and SecretomeP v2.0 [45,46].

**Figure 4:**
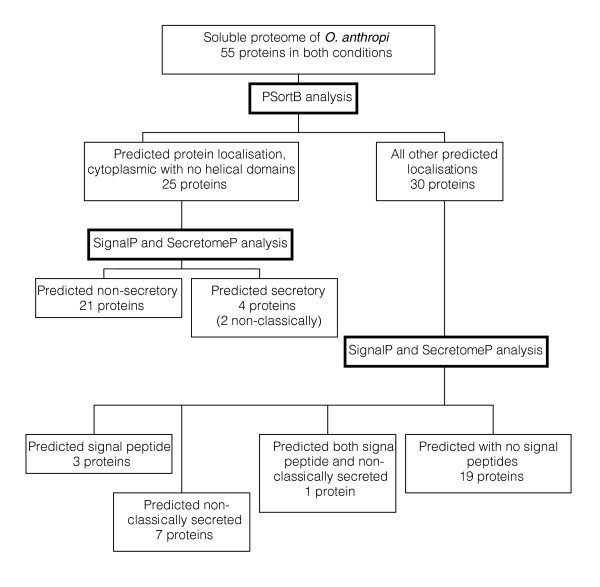
Overview of identified proteins from the soluble sub-proteome of *O. anthropi *present in both growth conditions. Cellular localization was predicted based upon the use of PSortB v2.0.4 [41,42], SignalP v3.0 [43,44], and SecretomeP v2.0 [45,46].

**Figure 5:**
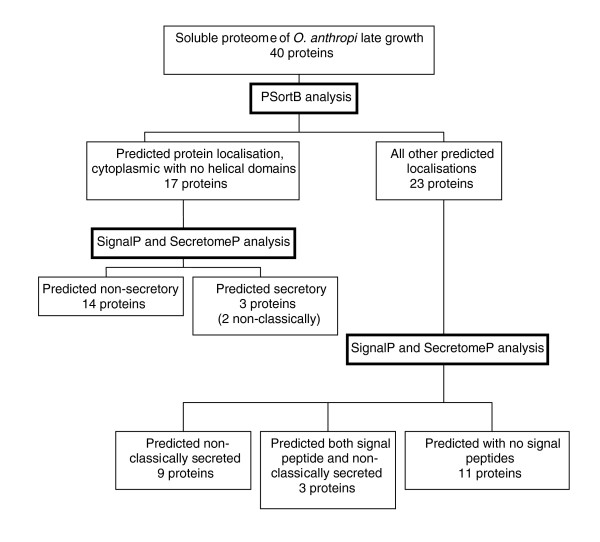
Overview of identified proteins from the soluble sub-proteome of *O. anthropi *present in late growth conditions. Cellular localization was predicted based upon the use of PSortB v2.0.4 [41,42], SignalP v3.0 [43,44], and SecretomeP v2.0 [45,46].

Sub-cellular localization prediction tools have been used for many years to identify those proteins that are retained by and exported from cells. They may also have uses in identifying possible diagnostic and therapeutic targets as well as providing information on the functionality of a protein [41]. In the current study a number of bioinformatics tools, including PSortB [41,42], SignalP [43,44] and SecretomeP [45,46] were utilized. These bioinformatics tools endeavor to assign a sub-cellular location for each protein. These tools use a set of descriptor rules and a variety of computational algorithms and networks to analyze a protein's amino acid composition in an attempt to identify known motifs or cleavage sites. The proteins identified in this study were separated into three groups and analyzed using the above bioinformatics tools. The groups were: those proteins only identified in early growth (bioinformatics search results can be seen in Table 1); those proteins found to be common to both growth conditions (bioinformatics search results can be seen in Table 2); and those proteins identified only at late growth phase (bioinformatics search results can be seen in Table 3). Overviews of the bioinformatic analysis on the proteins from the soluble sub-proteome of *O. anthropi *are shown for early growth (Figure 3), for both growth conditions (Figure 4) and for late growth (Figure 5).

Within the protein subset identified only in early growth, nine proteins were predicted to be secreted (26.5%), with six of those identified as possessing an amino-terminal signal peptide (Table 1); of those proteins common to both growth conditions, 15 were predicted to be secreted (27.3%), with five of those identified as possessing an amino-terminal signal peptide (Table 2); and of those identified only in late growth, 15 were predicted to be secreted (37.5%), with six of those identified as possessing an amino-terminal signal peptide (Table 3).

The subset of 17 proteins identified as possessing an amino-terminal signal peptide were further analyzed for the presence of lipobox, RR-motif, and signal peptide cleavage sites to allow assignment, where possible, to a particular secretion pathway [31,32] (Table 4). Of these 17 proteins, only seven had the required architecture that would allow them to be assigned to the Sec pathway (NCBI:17982453, 17984491, 17982015, 17982340, 17982154, 17983192 and 86283673). The remainder of the proteins, whilst containing the correct cleavage site for a signal peptide, did not, in fact, have the full amino-terminal architecture that would be required to allow us to classify them as secreted proteins [47,48]. This once again highlights the limitations of some of the present generation of bioinformatic tools, which presently are concentrated largely on motif-based predictors. This aptly demonstrates the absolute necessity of manual interpretation of results in order to gain any level of biological significance.

**Table 4:** Proteins identified within the soluble sub-proteome of *O. anthropi *containing predicted export signal peptides

Accesion no. (NCBI)	Function	Signal peptide
17982947	Methionyl-tRNA synthetase	MS*PLTNFFS*RA**YHA**
17983887	Dihydroxy-acid dehydratase	MKMPPYRSRTTTHGRNM**AGA**
17982453*	Hypothetical protein (immunoreactive 28 kDa omp)	MNTRASN*FLAASFSTIMLVGAFSL*P**AFA**
17983949	3-Deoxy-manno-oculosonate cytidylyltransferase	MVLLPPRKTARVGTRRKPVFLSQTC**ANG**
17984491*	Lipoprotein (ABC transporter substrate binding protein)	MSSVLSRYALTRRAGLK*ALLFTAAALTVGFASA*P**SHA**
27353255	Transriptional regulatory protein	MRAFTRFSY**SHS**
17982679	Protein translation elongation factor Tu	MCWRLSGSRTKRTT**AMA**
17982015*	Malate dehydrogenase	MRKETIMARNK*IALIGSGMIGG***TLA**
17982340*	Periplasmic dipeptide transport protein precursor	MGCARQAFPWRRTIMKFYQK*LLAATALVALMSGA***ASA**
17982205	Transketolase	MLCVPLPSGASSR**KAA**
17982141	ATP synthase F1, beta chain	MAKAATPKTTAA**AEA**
17982826	DNA-binding protein HU alpha	MPMNKNE**LVA**
17982154*	Leucine, isoleucine, valine, threonine and alanine binding protein	MGVPTMRKT*LFSGVALAAVIAF*GGS**AWA**
17983192*	General L-amino acid-binding periplasmic protein AAPJ precursor	MKKT*LMTGVLGAAALFGIA*SG**ASA**
17982682	50S ribosomal protein L11	MAKKVAGQLKLQVP**AGA**
17983290	Dihydrodipicolinate synthase	MLGVPSICFRSSRMLKGS**ITA**
86283673*	Dipeptide ABC transporter, substrate binding	MMITRLSRKFR*LLSAGAALSLLMMAA*PS**AFA**

Putative signal peptides were predicted as described by Tjalsma *et al*. [47] and Pugsley [48]. *Proteins likely to be secreted via the Sec pathway. Signal peptide: the hydrophobic H-domain is in italics; positively charged amino acids are underlined; the signal peptide cleavage sites comprise the last three amino acid residues and are in bold.

### Protein expression changes and pathway reconstruction

**Figure 6:**
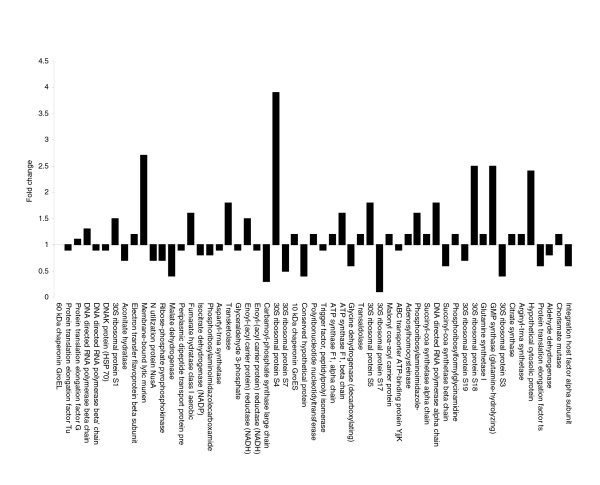
Differential expression profile of proteins common to both growth phases of *O. anthropi*. Fold changes of ≥1.5 or ≤0.5 are significant.

Utilizing the emPAI calculation for measuring protein abundance within our proteomic investigation allowed us to use the molar fraction percentage values for proteins common to both growth conditions; this enabled us to compare the fold change in protein expression that occurs under the two different conditions [37,49,50]. Two ranges are generally used in comparative proteomics to ascertain if the fold change in expression is significant. In the isobaric labeling technology iTRAQ™, a ≥20% change is considered significant and sufficient to take account of systematic errors; therefore, fold changes of ≥1.2 or ≤0.8 are significant, with a fold change value of 1 representing no difference in protein levels between the two states [51,52]. In the comparative two-dimensional PAGE technologies, a ≥50% change is considered significant and sufficient to take account of systematic errors; therefore, fold changes of ≥1.5 or ≤0.5 are significant, again with a fold change value of 1 representing no difference in protein levels between the two states [53,54]. The fold change in protein expression between proteins from the two growth conditions can be seen in Table 2. Taking the ≥20% cut-off value, 44 proteins significantly changed in expression; at the ≥50% cut-off value this is reduced to 19 proteins that significantly changed in expression between the two growth conditions. Utilizing the more stringent ≥50% value as a measure of differential protein expression, it can be seen that 11 proteins have much higher expression levels in the early growth condition and 6 have higher expression levels in the later growth condition (Figure 6).

Using the available genome sequence of *B. melitensis *16M, the closest relative of *O. anthropi*, and assuming a high degree of synteny between the genomes of these organisms, we investigated the genomic context of each gene found to be differentially expressed in this study. In this manner, we hoped that predicted transcriptional units for these proteins would be identified, thus elevating our functional understanding of the processes occurring within the organism.

Of the 30S ribosomal proteins identified as differentially expressed, all were predicted to be transcribed independently [55], and four (30S ribosomal proteins S3, S5, S7 and S17) were found within the same region of the *B. melitensis *16M genome. The reported differential expression of ribosomal proteins is not unusual in proteomics investigations; however, little information is available as to why certain component proteins of the 30S ribosome should be present at different levels. Of the remaining 12 proteins that were differentially expressed, the available *in silico *evidence suggests they are independently transcribed within the *B. melitensis *16M genome [55].

### Pathway identification

Previously, Djordjevic *et al*. [56] reported the necessity to identify within a proteomic study three enzymes present in a particular biochemical pathway in order to definitively state that such a pathway is present and active within the system under study. *Ergo*, in conjunction with the pathway reconstruction tool BioCyc [57], we have been able to identify the following pathways: superpathway of glycolysis, pyruvate dehydrogenase and TCA cycle (10 proteins) (Additional data file 1); superpathway of glyoxylate cycle (3 proteins) (Additional data file 1); fatty acid elongation (4 proteins) (Additional data file 2); *de novo *purine nucleotide biosynthesis (9 proteins) (Additional data file 3); arginine biosynthesis (3 proteins). Lying outside of our stringent rules for pathway identification but nonetheless worthy of note are two additional short pathways for which two out of four proteins (non-oxidative branch of the pentose phosphate pathway) and two out of three proteins (phenylalanine biosynthesis II) were identified in the current study.

At both time points it is clear, as would be expected, that central metabolic pathways such as the TCA cycle and fatty acid biosynthesis are active in *O. anthropi*. In addition, two key enzymes involved in the oxidative pentose phosphate pathway, transketolase and transaldolase, are also found. These enzymes are essential in the recycling of excess pentose phosphate, formed when there is high demand on NADPH_2_-dependent biosynthetic pathways. In addition, this pathway also provides intermediates for nucleotide biosynthesis, and indeed nucleotide biosynthetic pathways are also apparently active, as might be expected, at both sampling points [58]. In early phase growth, enzymes necessary for peptidoglycan and lipid A biosynthesis were specifically detected, presumably due to the high demand for new cell wall and outer membrane layer components at this growth point [59]. Whilst the enzymes essential for ribonucleotide biogenesis were present at both conditions, only in early phase growth was nucleoside diphosphate kinase, the key component for deoxyribonucleotide synthesis, detectable, indicative of a demand for components involved in DNA replication. In late phase growth, evidence for the activation of gluconeogenesis was found as a likely result of nutrient depletion, with expression of malate dehydrogenase upregulated by 2.4-fold, and the two enzymes phosphoenol pyruvate (PEP) carboxykinase and pyruvate phosphate dikinase, essential for the production of PEP from oxaloacetate and pyruvate, respectively, detectable for the first time. It is of note that this pathway is considered to be important for the establishment of host infection by certain bacteria, such as *Mycobacterium bovis *and *Xanthomonas campestris *[60,61]. Similarly, the presence of serine hydroxymethyltransferase, which converts glycine to L-serine prior to its potential conversion to pyruvate, was found only in late phase growth. Intriguingly, the enzymes involved in biosynthesis of the amino acids arginine, lysine and phenylalanine were also unique to late phase growth. Whilst this may reflect a cellular demand for these amino acids, it is of note that the co-product of arginine synthesis is fumarate, which has the dual role of being an intermediate in both the TCA cycle as well as in gluconeogensis.

**Figure 7:**
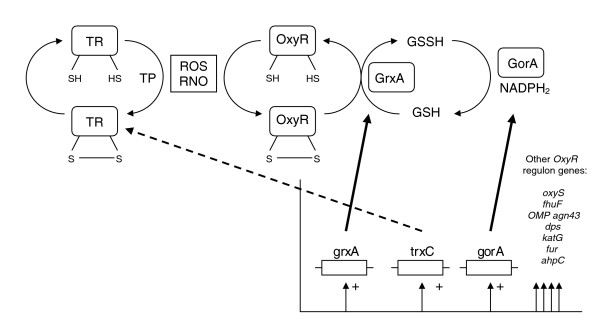
Proposed model for induction of the *OxyR *regulon in *O. anthropi*. Oxidized oxyR regulates expression of the *OxyR *regulon in response to oxidative and nitrosative stress, inducing *trxC*, *grxA*, *gorA *and other *OxyR *regulon genes. *ahpC*, alkyl hydroperodide reductase I; *fhuF*, ferric reductase; *fur*, ferric uptake repressor; GSSH/GSH, oxidized/reduced glutathione; GorA, glutathione reductase; GrxA, glutaredoxin A; *katG*, catalase (hydroperoxidase I); *OMP agn43*, outer membrane protein; *oxyS*, regulatory RNA; RNO, reactive nitrogen species; ROS, reactive oxygen species; TP, thiol peroxidase; TR, thioredoxin 2. (Adapted (with kind permission of Springer Science and Business Media) from Figure 2a [70].)

One additional feature of late phase growth is the presence of a number of stress response proteins, particularly those of importance in oxidative stress resistance. The proteins thioredoxin (TrxC), alkyl hydroperoxide reductase (AhpC) and thiol peroxidase have been reported to be important for the survival of pathogenic bacteria within a host organism [62,63]. Indeed, these proteins have also been detected in both transcriptomic and proteomic studies investigating the role of oxidative stress in a number of important pathogenic organisms that include *Escherichia coli*, *Candida albicans *and *Porphyromonas gingivalis *[64-68]. The *TrxC *and *AhpC *genes are subject to control by the *oxyR *regulon, which is induced in response to oxidative stress resulting from hydrogen peroxide and other free oxygen radicals. During this process, the regulatory protein oxyR becomes oxidized by these reactive oxygen species to form an intramolecular disulphide bond, thus allowing the activation of expression from *trxC*, *grxA*, *gorA *and hence other genes of the *OxyR *regulon (Figure 7). Glutathione is an essential element in the regulatory cycle of oxyR, which may explain the presence of N-methylhydantoinase in late phase growth, as it is an integral part of the γ-Glutamyl pathway, which is responsible for the generation of glutathione from 5-oxoproline. During oxidative stress thioredoxin is produced and in its reduced form it acts as an acceptor of oxygen radicals generated as a result of the catalytic activity of thiol peroxidase on H_2_O_2_, thus scavenging and detoxifying the damaging oxygen radicals and concomitantly forming H_2_O and thioredoxin disulphide [69]. Thioredoxins may also be involved in a cascade that triggers the transcription of other detoxifying genes, as they have also been shown to interact with DNA gyrase and thus influence a multitude of transcriptional responses in the cell by increasing or decreasing DNA supercoiling. This strongly suggests that the gyrase-mediated effect of thioredoxins on gene expression is a common redox-dependent signaling pathway in bacterial adaptation [70]. Intriguingly, AhpC has also been found to be an important conserved bacterial allergen that interacts with mammalian Toll-like receptors, specifically the MyD88 protein, thus activating innate and adaptive immune responses within the host [71].

## Conclusion

The popularity of 'identification' proteomics is evident from the abundant studies reported in the literature, and their contribution to our understanding of the diversity of protein expression in cells and organisms has been immense; however, these studies have clear limitations with regard to the amount of insight they can give on the function of a system. The trend within the proteomics community has, therefore, moved from this cataloguing approach towards the development of comparative and quantitative analyses that, as outlined in the present study, give greater insights into the functional processes occurring. Combining both of these techniques with rigorous data curation and interpretation, coupled with the vast array of bioinformatics tools available to the life scientist is the only way to ensure that these processes are accurately described such that meaningful data can be provided for the wider scientific community.

In this study we were able to identify distinct proteomic profiles associated with specific growth points for the emerging nosocomial pathogen *O. anthropi*. For those proteins common to both growth phases, the use of emPAI allowed a semi-quantitative analysis of protein expression to be made and it was possible to reconstruct core metabolic pathways functioning within this organism. It was also possible to infer unique functional and adaptive processes associated with specific growth phases and, therefore, gain a much deeper understanding of the physiology and metabolism of this pathogenic bacterium. Of particular interest was the identification of a number of protein products involved in oxidative stress response that are known to be regulated as part of the *oxyR *regulon and have previously been shown to be key in pathogen survival within host environments.

## Materials and methods

### Reagents

All reagents were purchased from Sigma-Aldrich (Poole, UK) with the exception of mass spectrometry grade water and acetonitrile, which were purchased from Romil (Cambridge, UK) and trypsin, which was purchased from Promega (Southampton, UK).

### Cell culture and growth conditions

*O. anthropi *UU551 was routinely maintained at 37°C on nutrient agar. Routine growth of the organism involved the inoculation of nutrient broth (50 ml in 250 ml Erlenmeyer flasks) with a loop of fresh, actively growing (16 h) culture from agar plates. Flasks were incubated aerobically at 37°C with orbital shaking at 200 rpm in an Innova™ 4230 refrigerated incubator shaker (New Brunswick Scientific, Edison, NJ, USA). Growth was monitored by the increase in culture attenuance at 600 nm.

### Protein extraction and quantification

*O. anthropi *cultures were harvested under two separate growth conditions, at early phase (D_600 _= 0.3) and at late phase (D_600 _= 1.2) growth, by centrifugation at 9000 × g for 10 minutes at 3-5°C. The cell pellet was weighed and resuspended in 10 mM phosphate-buffered saline (pH 7.8) at a ratio of 1 g cells to 2 ml buffer. The cells were then broken using sonication as described previously by Graham *et al*. [32]. The soluble proteome fraction was isolated by centrifugation of the homogenate at 25,000 × g for 30 minutes at 3-5°C (Beckman J2-HS, Beckman Instruments, Fullerton, CA, USA) followed by ultracentrifugation at 150,000 × g for 2 hours at 3-5°C (Beckman L8-M, Beckman Instruments) to sediment the insoluble fraction. The supernatant was decanted and stored frozen in 1 ml aliquots at -70°C until required. The total soluble protein content was measured using the Bradford assay [72].

### One-dimensional gel electrophoresis

An aliquot of the supernatant was diluted ten-fold with deionised water; 10 μl of this diluted sample was added to 10 μl Tris-Glycine SDS sample loading buffer (Invitrogen, Paisley, Renfrewshire, UK) and boiled for 5 minutes. The samples (20 μl; 100 μg total protein) were loaded onto a 1 mm thick Nu-Page 4-12% Bis-Tris gel (Invitrogen). SeeBlue™ Plus 2 (Invitrogen) was used as a protein molecular mass marker. The gel was electrophoresed, using MES SDS running buffer, in an X-Cell II mini gel system (Invitrogen) at 200 V, 120 mA, 25 W per gel for 35 minutes. Proteins were visualized using SimplyBlue™ Safestain (Invitrogen). The entire lane was excised from the gel and cut into nine fractions based on molecular mass as previously described by Graham *et al*. [35].

### In-gel tryptic digestion

Excised gel fractions were washed for 30 minutes in 200 mM NH_4_HCO_3_, pH 7.8 at 37°C. These fractions were then dehydrated by incubation for 30 minutes in 200 mM NH_4_HCO_3 _pH 7.8/MeCN (4:6 v/v) at 37°C, followed by rehydration for 30 minutes in 50 mM NH_4_HCO_3_, pH 7.8 at 37°C. Following incubation in 100% acetonitrile for 2 minutes, 0.1 μg trypsin in 50 mM NH_4_HCO_3_, pH 7.8 was added to each sample, which was then incubated overnight at 37°C. The supernatant was subsequently recovered into microcentrifuge tubes and a second peptide extraction from these gel pieces was carried out (0.1% trifluoroacetic acid (TFA) in 60% acetonitrile for 5 minutes). Peptide-containing liquid fractions were pooled, dried under vacuum and re-suspended in 20 μl 0.1% formic acid in 2% acetonitrile prior to storage at -70°C until required.

### Liquid chromatography-mass spectrometric analysis

Mass spectrometry was performed using a 3200 Q-TRAP Hybrid ESI Quadropole linear ion trap mass spectrometer, ESI-Q-q-Qlinear ion trap-MS/MS (Applied Biosystems/MDS SCIEX, Toronto, Canada) with a nanospray interface, coupled with an online Ultimate 3000 nanoflow liquid chromatography system (Dionex/LC Packings, Amsterdam, The Netherlands). A μ-Precolumn™ Cartridge (300 μm × 5 mm, 5 μm particle size) was placed prior to the C_18 _capillary column (75 μm × 150 mm, 3 μm particle size) to enable desalting and filtering. Both columns contained the reversed phase material PepMAP™ 100 (C_18 _silica-based) with a 100Å pore size (Dionex/LC Packings). The elution buffers used in the gradient were Buffer A (0.1% formic acid in 2% acetonitrile) and Buffer B (0.1% formic acid in 80% acetonitrile). The nanoLC gradient used was 60 minutes in length: 0-55% B in 45 minutes, 10 minutes at 90% B followed by 5 minutes at 100% A. The flow rate of the gradient was 300 nlmin^-1^. The detector mass range was set at 400-1,800 *m/z*. MS data acquisition was performed in positive ion mode. During MS acquisition, peptides with 2+ and 3+ charge states were selected for fragmentation.

### Database searching, protein identification and PROVALT analysis

Protein identification was carried out using an internal MASCOT server (version 1.9; Matrix Science, London, UK) searching against the bacteria sub-set of the MSDB database (latest version at the time of processing). Peptide tolerance was set at ± 1.2 Da with MS/MS tolerance set at ± 0.6 Da and the search set to allow for one missed cleavage. In order to expedite the curation of the identified protein list from MASCOT, the result files were re-analyzed against an extracted database comprising eleven α-proteobacterial genome databases downloaded from NCBI using the heuristic method known as the protein validation tool PROVALT [36]. This automated program takes large proteomic MS datasets and reorganizes them by taking multiple MASCOT results and identifying those peptides that match. Redundant peptides are removed and related peptides are grouped together associated with their predicted matching protein; thus, the program dramatically reduces this portion of the curation process. For identification purposes the minimum peptide length was set at 6 amino acids, the minimum peptide MOWSE score was set at 25 and the minimum high quality peptide MOWSE score was set at 40. PROVALT also uses peptide matches from a random database (in this case the extracted α-proteobacterial database was randomized) to calculate false-discovery rates (FDRs) for protein identifications as previously described by Weatherley *et al*. [36]. Briefly, identifications from searching the normal and random databases are used to calculate the FDRs and set score thresholds and thus identify as many 'actual' proteins as possible while encountering a minimal number of false-positive protein identifications. Rather than calculate error rates at the peptide level, the FDR calculations employed by PROVALT provide a reasonable balance between the number of correct and incorrect protein assignments. In this study the FDR was set at 1%, meaning that 99% of the reported proteins identified should be correct.

### Protein quantification and abundance measurements

Proteins within the two growth conditions were quantified utilizing emPAI [37,49,50]. This method allows the quantification of individual identified proteins by utilizing database and Mascot output information, in order to give an emPAI value. The emPAI value can then be used to estimate the protein content within the sample mixture in molar fraction percentages. Also, the fold change in expression levels of proteins identified under both growth conditions can be estimated, allowing further insights into cellular processes.

### Pathway reconstruction

Pathways were reconstructed utilizing the BioCyc database [57], a collection of 160 pathway/genome databases for most eukaryotic and prokaryotic species whose genomes have been completely sequenced. The BioCyc collection provides a unique resource for computational systems biology by enabling global and comparative analyses of genomes and metabolic networks. Identified proteins can be entered into the database and searched against specific species, thus allowing scientists to visualize combinations of gene expression maps of these organisms and thus reconstruct pathways that are present.

### Bioinformatics

PSORTb version 2.0.4 [41,42] was used for the prediction of bacterial protein subcellular localization. SignalP 3.0 [43,44] was used to predict the presence and location of signal peptide cleavage sites in amino acid sequences, for classically secreted proteins. SecretomeP 2.0 [45,46] was used for the prediction of non-classical protein secretion (that is, protein secretion that is not triggered by signal peptides).

## Additional data files

The following additional data are available with the online version of this paper. Additional data file 1 is a figure illustrating the superpathway of glycolysis, pyruvate dehydrogenase, TCA and the superpathway of the glyoxylate cycle. Additional data file 2 is a figure illustrating fatty acid elongation. Additional data file 3 is a figure illustrating *de novo *purine nucleotide biosynthesis. Within these pathways proteins unique to the early growth phase are boxed in green, those identified in both growth conditions are boxed in blue and those unique to the late growth phase are boxed in yellow.

## Supplementary Material

Additional data file 1 Proteins unique to the early growth phase are boxed in green, those identified in both growth conditions are boxed in blue and those unique to the late growth phase are boxed in yellow. Click here for file

Additional data file 2 Proteins unique to the early growth phase are boxed in green, those identified in both growth conditions are boxed in blue and those unique to the late growth phase are boxed in yellow. Click here for file

Additional data file 3 Proteins unique to the early growth phase are boxed in green, those identified in both growth conditions are boxed in blue and those unique to the late growth phase are boxed in yellow. Click here for file
