# Supplementary figures and images for: A semi-quantitative GeLC-MS analysis of temporal proteome expression in the emerging nosocomial pathogen Ochrobactrum anthropi
Source: Genome Biol. 2007 Jun 13;8(6):R110. doi: 10.1186/gb-2007-8-6-r110 (PMC2394761; doi:10.1186/gb-2007-8-6-r110)

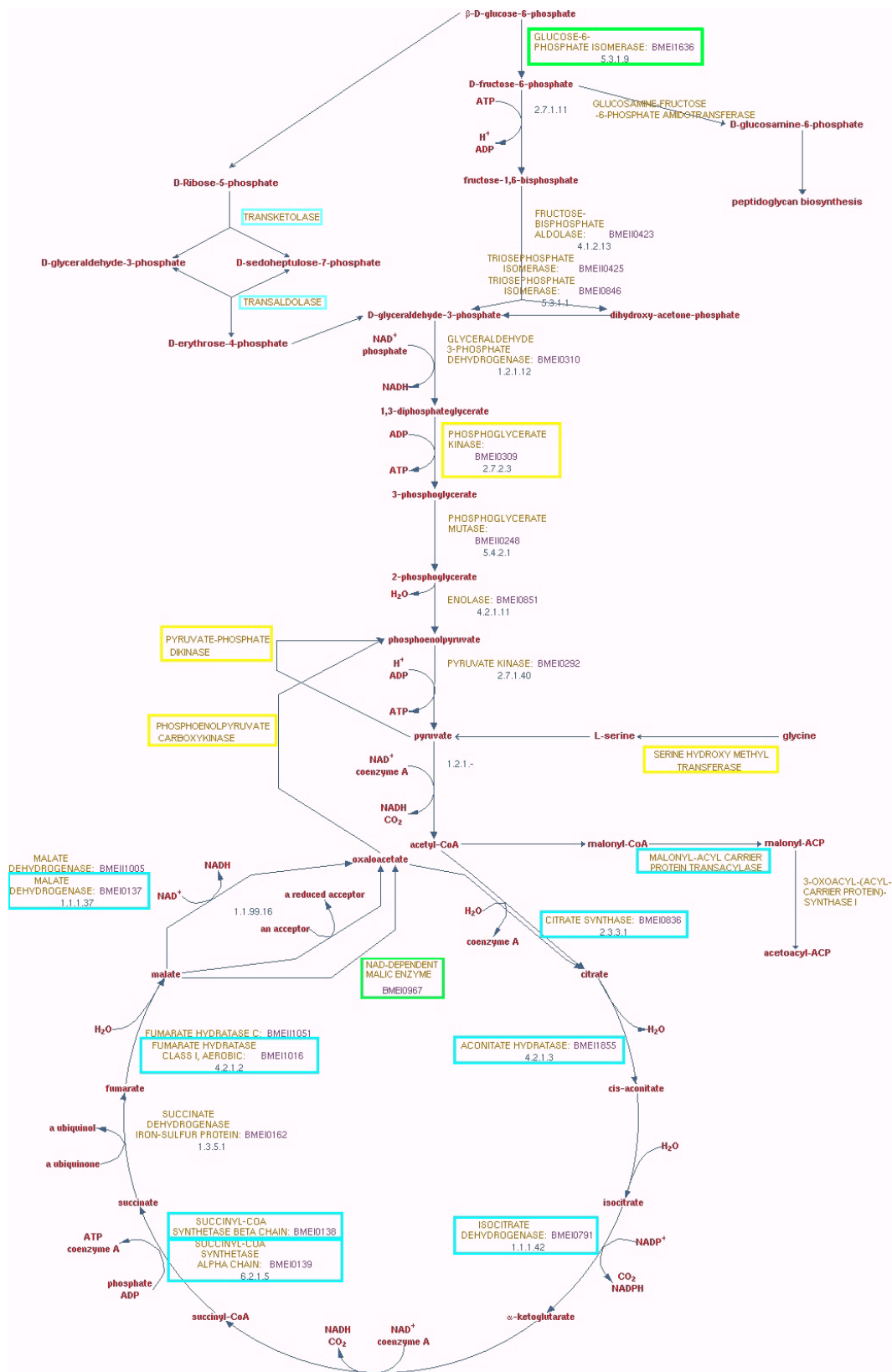

**SUPPLEMENTAL PATHWAY 1: GLYCOLYSIS, PYRUVATE DEHYDROGENASE AND TCA CYCLE.**

Supplement: Additional data file 1 — Proteins unique to the early growth phase are boxed in green, those identified in both growth conditions are boxed in blue and those unique to the late growth phase are boxed in yellow. [file gb-2007-8-6-r110-S1.pdf]

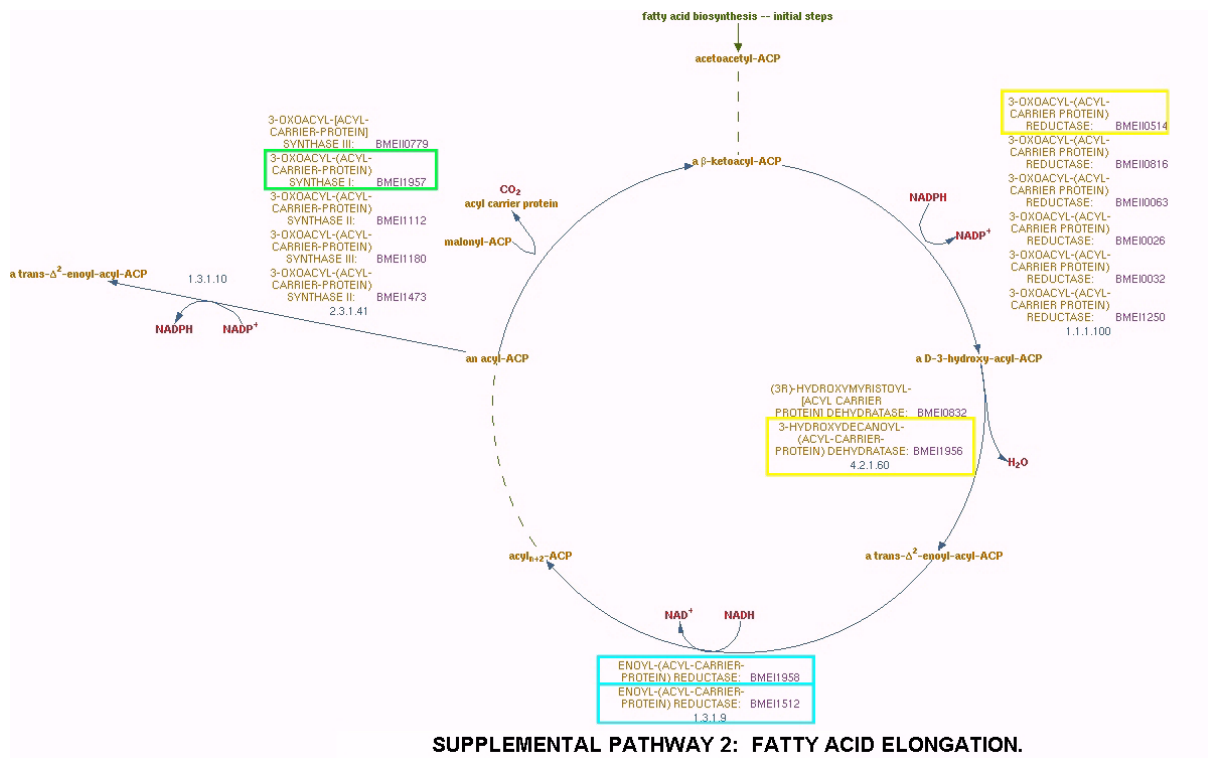

Supplement: Additional data file 2 — Proteins unique to the early growth phase are boxed in green, those identified in both growth conditions are boxed in blue and those unique to the late growth phase are boxed in yellow. [file gb-2007-8-6-r110-S2.pdf]
